# Double Encapsulation of Resveratrol and Doxorubicin in Composite Nanogel—An Opportunity to Reduce Cardio- and Neurotoxicity of Doxorubicin

**DOI:** 10.3390/gels10110699

**Published:** 2024-10-28

**Authors:** Lyubomira Radeva, Yordan Yordanov, Ivanka Spassova, Daniela Kovacheva, Virginia Tzankova, Krassimira Yoncheva

**Affiliations:** 1 Faculty of Pharmacy, Medical University of Sofia, 1000 Sofia, Bulgaria; yyordanov@pharmfac.mu-sofia.bg (Y.Y.); vtzankova@pharmfac.mu-sofia.bg (V.T.); 2 Institute of General and Inorganic Chemistry, Bulgarian Academy of Sciences, 1113 Sofia, Bulgaria; ispasova@svr.igic.bas.bg (I.S.); didka@svr.igic.bas.bg (D.K.)

**Keywords:** composite nanogel, resveratrol, doxorubicin, chitosan, albumin, hydroxypropyl-β-cyclodextrin, cardiotoxicity, neurotoxicity

## Abstract

The simultaneous encapsulation of drugs into nanosized delivery systems could be beneficial for cancer therapies since it could alleviate adverse reactions as well as provide synergistic effects. However, the encapsulation of hydrophobic drugs into hydrophilic nanoparticles, such as nanogels, could be challenging. Therefore, innovative technological approaches are needed. In this research, a composite nanogel system was prepared from chitosan, albumin, and hydroxypropyl-β-cyclodextrin for co-delivery of the hydrophilic anticancer drug doxorubicin and hydrophobic antioxidant resveratrol. The nanoparticles were characterized using dynamic light scattering and found to have a hydrodynamic diameter of approx. 31 nm, narrow size distribution (PDI = 0.188), positive ƺ-potential (+51.23 mV), and pH-dependent release of the loaded drugs. FTIR and X-ray analyses proved the successful development of the composite nanogel. Moreover, the double-loaded system showed that the loading of resveratrol exerted protection against doxorubicin-induced toxicity in cardioblast H9c2 and neuroblast SH-SY5Y cells. The simultaneous loading did not influence the cytostatic effect of the antitumor agent in lymphoma L5178Y and L5178MDR cell lines.

## 1. Introduction

Developing new strategies to improve the application of drugs and to enhance the effectiveness of different therapies is a widely researched topic nowadays. Nanosized drug delivery systems offer a huge number of advantages that could be beneficial for modern medicine. Nanogels are a type of hydrophilic nanoparticles with deformable and elastic structures that provide the possibility of passive targeting and easier crossing through biological barriers [1,2,3]. They are also highly stable structures and have the ability to protect the incorporated active substances from internal and external factors [4]. Moreover, the biodegradability and biocompatibility of nanogel particles make them appropriate for parenteral administration [5]. Their hydrophilicity and small size lead to decreased levels of opsonization and formation of protein corona, resulting in longer blood circulation [6]. The possibility of developing nanogels with polymers containing ionizable functional groups, such as amines and carboxylic acids, allows for achieving pH-dependent release, making them suitable carriers for anticancer therapies [7]. For instance, Manzanares-Guevara et al. developed smart N,N′-diethylaminoethyl methacrylate-poly(ethyleneglycol) methacrylate nanogels, which had no toxicity in vitro and in vivo [8]. The system was stable in physiological conditions for 30 days and improved the anticancer activity of curcumin in vitro. Gelatin-hydroxyapatite nanogel provided a pH-dependent release of curcumin (more pronounced in an acidic medium) and improved cellular uptake and anticancer activity of the drug in human lung cancer cells A549 [9]. The loading of curcumin in chondroitin sulfate nanogel also resulted in improved cellular uptake in breast cancer MCF-7 cells [10]. As mentioned, the encapsulation of unstable drugs into nanogels could ensure their protection. For example, the photostability of doxorubicin has been significantly increased via encapsulation into citric acid-pentane-1,2,5-triol and chitosan-albumin nanogels [11,12].

The simultaneous delivery of drugs, especially for anticancer therapy, is gaining significant attention nowadays. This approach could result in a reduction of the toxic effects of the loaded drugs, overcoming multidrug resistance, and synergistic anticancer action [13]. Synergistic antitumor effect in resistant breast cancer MCF-7/ADR cells and in vivo in murine bearing MCF-7/ADR cells model was achieved via double-loading of doxorubicin and baicalein into nanostructured lipid carriers [14]. Similarly, Amjadi et al. encapsulated doxorubicin and betanin simultaneously into pH-responsive gelatin-based nanoparticles and enhanced the antitumor activity in MCF-7 cells [15]. The double incorporation of docetaxel and resveratrol into methoxyl poly(ethylene glycol)-poly(d,l-lactide) micelles also led to decreased IC_50_ in MCF-7 cells [16]. Camptothecin and curcumin were loaded into chitosan-coated poly(lactic-co-glycolic acid) nanoparticles, resulting in a synergistic anticancer effect in colon adenocarcinoma Colon-26 cells [17]. Furthermore, the co-delivery of anticancer drugs with natural antioxidants could alleviate the adverse effects. For instance, the simultaneous encapsulation of doxorubicin, docosahexaenoic acid, and α-tocopherol succinate into nanostructured lipid carriers resulted in ameliorated heart and liver toxicity of doxorubicin and reduced mortality in mice [18]. Moreover, the systems showed synergistic anticancer effects in vitro in breast cancer 4T1 cells and in vivo in 4T1 tumor-bearing mice. The co-encapsulation of doxorubicin and silybin into distearoylphosphatidylethanolamine–polyethylene glycol–cholic acid-modified liposomes decreased cardiotoxicity of doxorubicin in cardioblast H9c2 cells [19]. The proliferation of hepatocellular cancer HepG2 cells and migration of HCC97H cells was also inhibited by the system. Furthermore, the authors observed decreased accumulation in heart tissue and increased accumulation in liver tissue.

Despite the significant advantages of hydrophilic nanogels, in some cases, the incorporation of hydrophobic drugs into such structures is problematic [6]. The homogeneous distribution of hydrophobic substances into hydrogel matrices is limited, leading to low loading efficiency [20,21]. Therefore, new approaches for overcoming these drawbacks are needed. Developing composite nanoparticles consisting of two or more different nanoscale components could be considered an appropriate strategy for improving the loading of hydrophobic drugs into hydrophilic systems as well as for providing an opportunity for double-loading. For instance, zein-carboxymethyl cellulose composite nanoparticles were developed for the co-delivery of quercetin and resveratrol [22]. Since both drugs are hydrophobic, they were first encapsulated into a zein core, and then the system was coated with cellulose to increase stability. The hydrophilic doxorubicin and hydrophobic hydroxycamptothecin were simultaneously encapsulated into polydopamine-upconversion nanoparticle-mesoporous silica yolk-shell composite nanoparticles [23]. Thus, by developing composite nanogel systems, the advantages of nanogels, such as hydrophilicity and elasticity, as well as achieving high encapsulation efficiency of hydrophobic substances, could be combined.

The aim of this study was to develop a composite nanogel system that could enable the encapsulation of drugs with different water affinity, particularly doxorubicin and resveratrol. Since doxorubicin hydrochloride possesses serious adverse effects such as cardiotoxicity [24] and neurotoxicity [25], the simultaneous encapsulation with natural substances with antioxidant and anti-inflammatory effects (e.g., resveratrol) could be a suitable strategy to cope with these problematic issues. Thus, resveratrol was included in hydroxypropyl-β-cyclodextrin complex that was further simultaneously encapsulated with doxorubicin into chitosan-bovine serum albumin nanogel. The system was characterized via dynamic light scattering (DLS), transmission electron microscopy (TEM), infrared spectroscopy (IR), X-ray, and in vitro dissolution analyses. The ability of the system to reduce the cardiotoxicity and neurotoxicity of doxorubicin was evaluated in vitro in cardioblast H9c2 and neuroblast SH-SY5Y cells. Moreover, the cytostatic effects of the system were tested in lymphoma L5178Y and L5178MDR cells.

## 2. Results and Discussion

### 2.1. Preparation of Double-Loaded with Doxorubicin and Resveratrol Complex Composite Chitosan-Albumin Nanogels

**Figure 1:**
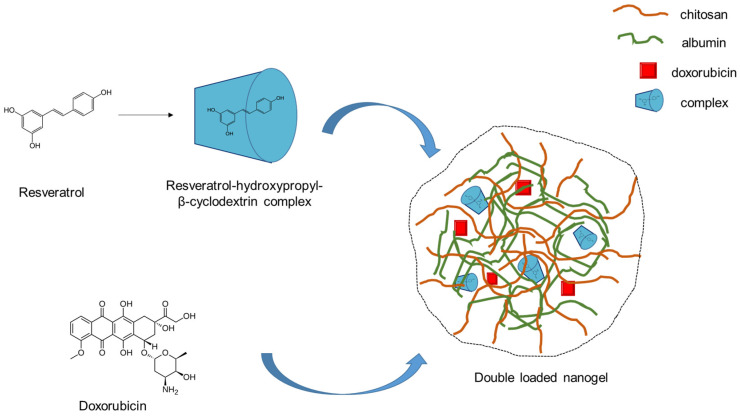
Schematic presentation of the preparation process of double-loaded with doxorubicin and resveratrol composite chitosan-albumin nanogel.

The double incorporation of substances with different water affinity into one nanosized delivery system could provide many advantages, but it is still a challenge [26,27,28]. Moreover, the loading of hydrophobic substances into hydrophilic systems such as nanogels requires technological approaches [29]. Resveratrol is known to possess low aqueous solubility (50 µg/mL) and high lipophilicity (logP = 3.1) [30]. Therefore, we applied a strategy for obtaining a composite nanosystem that will provide the successful encapsulation of both doxorubicin and resveratrol. First, a complex between resveratrol and hydroxypropyl-β-cyclodextrin at a ratio of 1:10 was developed via the solvent evaporation method [31]. Therefore, the double-loaded nanogel particles were prepared via electrostatic gelation and heating by modifying a previously reported procedure [11] (Figure 1). The encapsulation efficiency and loading degree of doxorubicin and resveratrol were 73.3% and 516 µg/mL and 97.8% and 324 µg/mL, respectively. These high values for the hydrophobic resveratrol confirmed that the developed approach was successful. For instance, the encapsulation efficiency for resveratrol in hydrophilic systems was found to be approx. 71% in alginate nanoparticles [32], 71–75% in sericin nanoparticles [33], and approx. 67% in chitosan nanoparticles [34]. Thus, the complexation of the drug in the current study led to improved loading into the hydrophilic nanogel. Furthermore, this approach allowed high loading efficiency without using organic solvents, which have potential toxic effects.

### 2.2. Characterization of the Composite Nanogel

**Figure 2:**
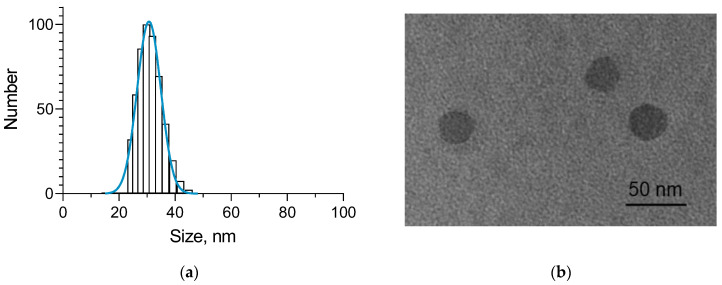
Histogram of the mean diameter (**a**) and TEM image (**b**) of double-loaded with doxorubicin and resveratrol nanogel particles.

The double-loaded nanogel particles were examined via dynamic light scattering (DLS) and transmission electron microscopy (TEM). The analyses revealed that the nanoparticles were characterized with a small average size of approx. 31 nm, narrow size distribution, spherical shape, and positive zeta potential (Figure 2a,b, Table 1). The zeta potential of the system increased after the loading process. Since the positive charge of the surface of the empty nanogel was a result of the free amino groups of chitosan, probably more of these groups did not react with the carboxyl groups of albumin when the complex was added. A possible explanation could be the interaction between the cyclodextrin and albumin, leading to complexation and occupying the protein’s functional groups [35]. Moreover, a zeta potential in the range of 30–60 mV absolute value could provide excellent stability due to the repulsion between the nanoparticles and avoiding agglomeration [36].

**Figure 3:**
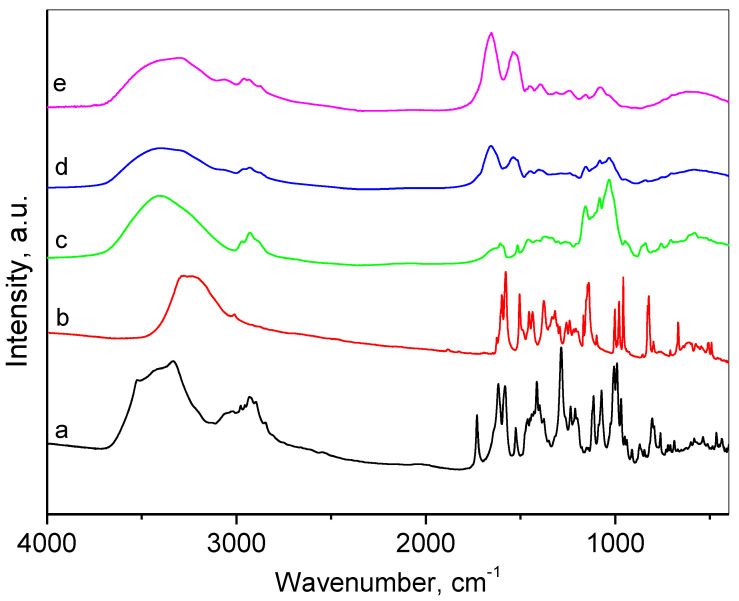
FTIR spectra of: (a) doxorubicin (DOX), (b) trans-resveratrol (RES), (c) resveratrol complex with HP-β-CD, (d) doxorubicin and resveratrol loaded nanogel (DOX/RES-NG), (e) empty nanogel (NG).

Figure 3 presents the FTIR spectra of initial drugs, complex with resveratrol and nanogel. The spectrum of doxorubicin and resveratrol coincide with those reported in the literature [37,38]. The characteristic bands of pure doxorubicin appearing at 1733 cm^−1^ indicated the presence of C=O bonds, and the vibrations of phenol rings at 1615 cm^−1^ and 1585 cm^−1^ were visible. The lines at 1418 cm^−1^, 1282 cm^−1^, and 989 cm^−1^ were due to the vibrations of N–H, C–C, and C–OH bonds, respectively. The following bands considered as characteristic of resveratrol were registered at 1603 cm^−1^, ascribed to aromatic C=C bond stretching, 1582 cm^−1^ for olefinic C=C stretching, and 967 cm^−1^ typical for trans-olefinic bond [39]. The disappearance of the line at 967 cm^−1^ in the FTIR spectrum of resveratrol complex with HP-β-CD suggests the successful complex formation [31]. The spectrum of the empty nanogel presented a set of bands typical for both chitosan at 1081 cm^−1^ and 1021 cm^−1^ for stretching vibrations of C–O–C and P=O bonds [40], and albumin at 1651 cm^−1^ for C=O stretching, amide I, -NH bending at 1538 cm^−1^, amide II, and 1395 cm^−1^ for C–N stretching [41]. The FTIR spectrum of DOX/RES-NG consisted of the main characteristic bands of resveratrol complex, doxorubicin, and the nanogel particles. The successful inclusion of doxorubicin was proved by the presence of 989 cm^−1^ shoulder band, and the encapsulation of the resveratrol complex into the nanogel was suggested by the increase of the intensities of the peaks in 1157 cm^−1^, 1084 cm^−1^, and 1032 cm^−1^ due to vibrations of C–O and C–O–C bonds.

**Figure 4:**
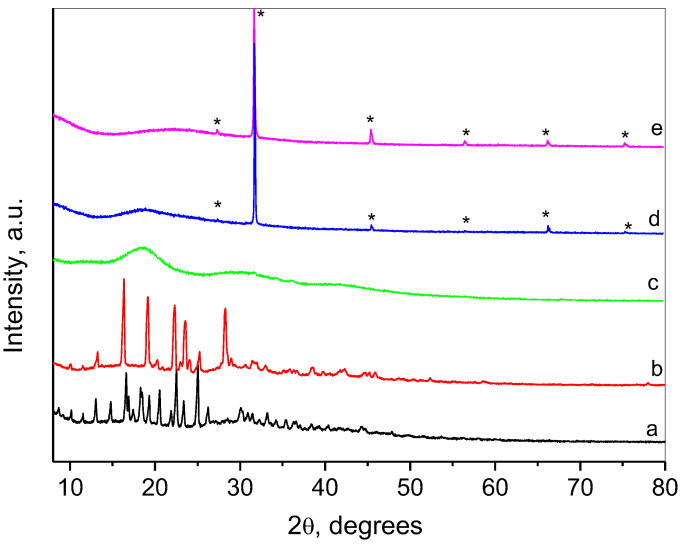
Powder XRD patterns of (a) doxorubicin (DOX), (b) trans-resveratrol (RES), (c) resveratrol complex with HP-β-CD, (d) doxorubicin and resveratrol loaded nanogel (DOX/RES-NG), (e) empty nanogel (NG).

Collected XRD patterns of the drugs, complex, and nanogels are shown in Figure 4. The pattern of doxorubicin consisted of a number of sharp, intense peaks revealing the high crystalline nature of the drug [37,42]. Resveratrol is also highly crystalline, with typical peaks corresponding to the crystal structure of the trans-form of the drug [43]. The pattern of resveratrol complex with HP-β-CD comprised three broad humps that showed successful interaction between the two components [31,44,45]. A typical amorphous compound diffraction pattern was observed for the empty nanogel (NG) with one very broad hump centered at 22.5° 2θ. The lines of crystalline NaCl (marked with asterisks in the diffraction patterns) were also registered using the preparation method. The pattern of the nanogel loaded with both drugs (DOX/RES-NG) consisted of an amorphous broad peak with a center coinciding with the first peak of the resveratrol complex. The disappearance of the sharp peaks of doxorubicin at this stage indicated its presence in a molecular form in the final DOX/RES-NG.

### 2.3. In Vitro Release Studies

**Figure 5:**
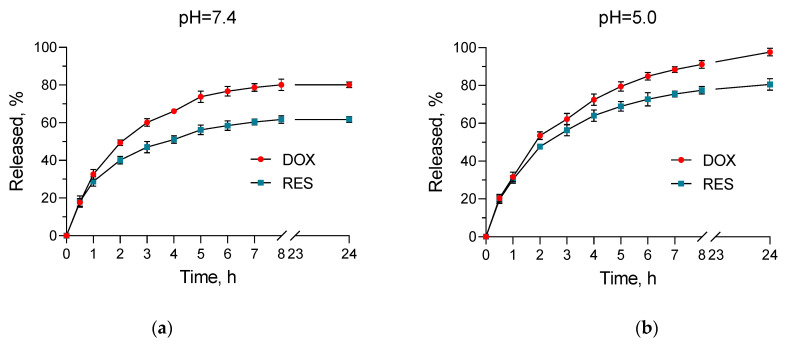
In vitro release profiles of doxorubicin (DOX) and resveratrol (RES) from the nanogel system in media with pH = 7.4 (**a**) and 5.0 (**b**). Mean ± SD (*n* = 3).

The pH of the extracellular medium surrounding healthy cells is around 7.4, while the extracellular pH value for cancer cells could decrease to approx. 6.0 [46]. Furthermore, the pH value in the endosomes and lysosomes is around 4.5 [47]. Therefore, we conducted in vitro dissolution tests in media with neutral (7.4) and slightly acidic pH (5.0). The tests showed a pH-dependent release. There was a higher amount of both drugs released in the medium with pH = 5.0 compared to the medium with pH = 7.4 (Figure 5). In particular, approx. 98% of doxorubicin was released in the acidic medium vs. around 80% in the neutral one. For resveratrol, these values were approx. 81 vs. 62%, respectively. This pH-dependent release manner of both doxorubicin and resveratrol draws attention to the system’s ability to provide specific delivery of the encapsulated drugs. The main reason for this is probably the solubility of chitosan, which is more pronounced in an acidic medium [48,49]. Similarly, more pronounced release from chitosan nanoparticles in such a slightly acidic medium was observed for different active substances such as tamoxifen [50], *Cinnamomum zeylanicum* essential oil [51], curcumin [52], and oxaliplatin [47]. Thus, the ability of the developed nanogel system to achieve pH-dependent release of the encapsulated drugs, as well as the small size, could enable the targeting of cancer cells, the efficiency of therapies, and the protection of healthy tissues.

Moreover, the release mechanism for doxorubicin and resveratrol was studied by fitting the data until the 8th hour by applying zero-order, first-order, and Higuchi mathematical models [53]. The results from the analyses are presented in Table 2 and Table 3. For doxorubicin in the medium with pH = 7.4, the highest coefficient (r^2^) was calculated for the Higuchi model, meaning that the process is controlled via diffusion through the polymer matrix. The greatest coefficient (r^2^) for doxorubicin in the neutral medium and for resveratrol in both acidic and neutral medium was found for first-order kinetics. Therefore, in these cases, the release of the drugs depended on their remaining concentration in the nanogel matrix.

### 2.4. Cytotoxicity Studies

**Figure 6:**
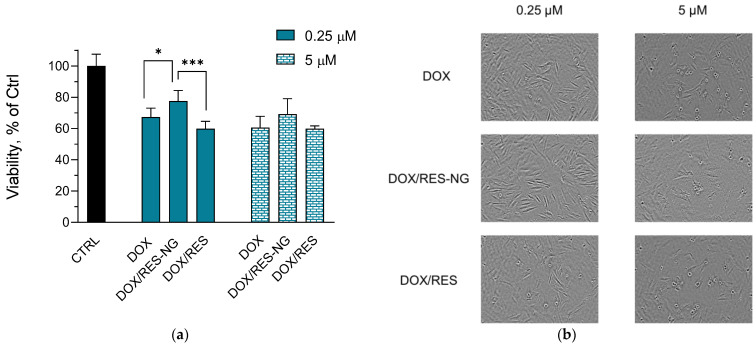
In vitro cytotoxicity (**a**) and digital images (**b**) of H9c2 cells treated with doxorubicin (DOX, positive control) at 0.25 or 5 µM, double-loaded nanogels (DOX/RES-NG) and referent solutions of doxorubicin and resveratrol (DOX/RES) at 0.25 or 5 µM concentration of both drugs; CTRL—negative control; Mean ± SD (*n* = 3). * *p* < 0.05, *** *p* < 0.001 between cell groups.

One of the main adverse effects of doxorubicin in anticancer therapies is its cardiotoxicity [54]. The induced oxidative stress by the antitumor drug is one of the reasons for this toxicity [55]. Therefore, the simultaneous delivery of doxorubicin and antioxidants, such as resveratrol, could be considered an appropriate strategy to ameliorate this toxicity. Furthermore, the advantages of the nanogel systems could additionally reduce the adverse effect via passive targeting. Taking this into consideration, we examined the potential of the double-loaded system to have a protective effect on H9c2 cardioblasts. First, the empty nanogel particles did not show any toxic effects on the cells. The in vitro test revealed that the simultaneous encapsulation of both substances is associated with decreased cardiotoxicity of doxorubicin (Figure 6a,b). In particular, the double-loaded nanogel showed a significant protective effect at 0.25 µM concentration of both drugs. At the higher concentration of 5 µM, a tendency for protection was also observed when the cells were treated with the encapsulated substances. On the contrary, the referent solutions of the non-encapsulated drugs showed cytotoxicity similar to that of pure doxorubicin in both concentrations. This is also confirmed by the microscopic images of the cells. Namely, the typical morphology of apoptotic cells (shrinkage and more prominent phase contrast halo) [56,57] is more common in the images of the cells treated with doxorubicin or with the referent solution in comparison with the cells treated with the double-loaded nanogels. Thus, the encapsulation of resveratrol seems to enable its cardioprotective effect, whereas its non-encapsulated form did not exert such an effect on the cardioblasts.

**Figure 7:**
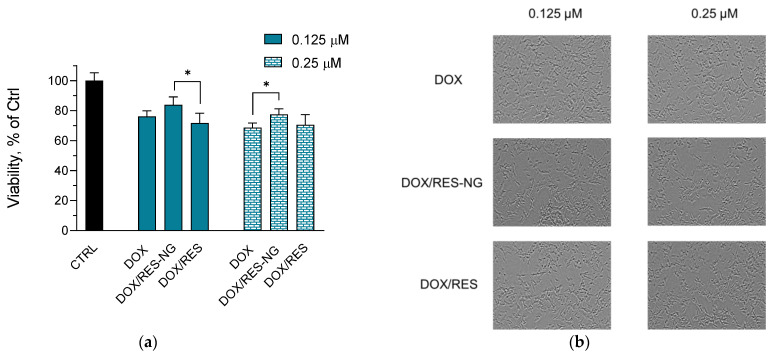
In vitro cytotoxicity (**a**) and digital images (**b**) of SH-SY5Y cells treated with doxorubicin (DOX, positive control) at 0.25 or 5 µM, double-loaded nanogel (DOX/RES-NG) and referent solutions of doxorubicin and resveratrol (DOX/RES) at 0.25 or 5 µM concentration of both drugs; CTRL—negative control; Mean ± SD (*n* = 3). * *p* < 0.05 between cell groups.

Another adverse effect of doxorubicin is its neurotoxicity [58]. Oxidative stress, particularly lipid peroxidation, is considered to be a part of the mechanisms responsible for this adverse effect [59]. This was a prerequisite for evaluating the potential of the simultaneous delivery of doxorubicin, along with resveratrol, to alleviate neurotoxicity in vitro. The in vitro assays on neuroblastoma SH-SY5Y cells revealed that the empty nanogel did not have any cytotoxic effects. Similar to the cardioblasts, only the double-loaded nanogel showed protective effects against doxorubicin-induced neurotoxicity (Figure 7a,b). At 0.125 µM concentration, we observed a tendency for protection, while at 0.25 µM, there was a significant protective effect vs. the doxorubicin-treated group. The referent solution of doxorubicin and resveratrol did not show any protective effects. This could be confirmed from the microscopic images, where the morphological changes such as shrinkage and fragmentation are observed more often for the cells treated with doxorubicin and the referent solution, compared to the cell groups treated with the loaded nanogels [56,57].

**Figure 8:**
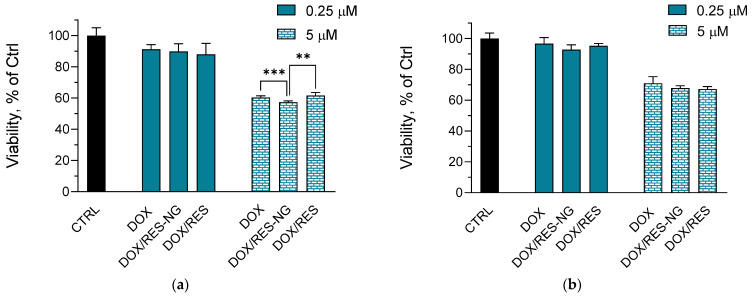
In vitro cytotoxicity of L5178Y (**a**) and L5178 MDR (**b**) cells treated with doxorubicin (DOX) at 0.25 or 5 µM, double-loaded nanogel (DOX/RES-NG) and referent solutions of doxorubicin and resveratrol (DOX/RES) at 0.25 or 5 µM concentration of both drugs; Mean ± SD (*n* = 3). ** *p* < 0.01, *** *p* < 0.001 between cell groups.

The simultaneous delivery of anticancer drugs with antioxidants could influence the cytostatic activity of the antitumor agent. However, resveratrol is known to possess antitumor effects via its prooxidant activity, induction of autophagy, and apoptosis [60,61]. Therefore, we evaluated the cytotoxic effects of the double-loaded nanogel in lymphoma L5178Y and multidrug-resistant L5178MDR cell lines. The empty nanogel did not reduce the viability of the cell lines. As can be seen in Figure 8, the double encapsulation of doxorubicin and resveratrol did not reduce the cytostatic effect of the antitumor drug in both L5178Y and L5178MDR cell lines. Moreover, at 5 µM concentration of both drugs, there was a decrease in the cell viability of L5178Y cells when they were only treated with the loaded nanogel (Figure 8a).

Thus, the double encapsulation of doxorubicin and resveratrol in the nanogel system resulted in reduced cardiotoxicity and neurotoxicity, especially at 0.25 µM concentration. The system did not decrease the cytostatic effect of doxorubicin in lymphoma cells; besides, at 5 µM concentration, there was an enhanced cytostatic effect in L5178Y cells. This is probably due to the concentration-dependent prooxidant effects of resveratrol [62].

## 3. Conclusions

Composite nanogel particles were successfully developed from chitosan, bovine serum albumin, and hydroxypropyl-β-cyclodextrin. The composite system enabled high encapsulation efficiency of hydrophilic doxorubicin (73.3%) and hydrophobic resveratrol (97.8%). The double-loaded nanogel particles were characterized by small size of approximately 30 nm, narrow size distribution, and pH-dependent release of both drugs, which was faster in medium with pH = 5.0. Therefore, we consider the double-loaded nanogel appropriate for application in cancer treatment. Preliminary in vitro studies revealed the potential of the double-loaded nanogel to alleviate cardiotoxicity and neurotoxicity related to doxorubicin, as well as to maintain its anticancer activity in lymphoma cells.

## 4. Materials and Methods

### 4.1. Materials

Chitosan (Mv 110,000–150,000), bovine serum albumin (fraction V), hydroxypropyl-β-cyclodextrin, doxorubicin hydrochloride, trans-resveratrol, Dulbecco’s Modified Eagle’s Medium, Roswell Park Memorial Institute 1640 Medium, McCoy’s 5A Medium, fetal bovine serum (FBS), L-glutamine and colchicine were obtained from Sigma Chemical Co. (Germany). Neutral red and Resazurin were supplied by Thermo Fisher Scientific (Waltham, MA, USA). The cardioblast cell line H9c2 and the neuroblastoma SH-SY5Y were purchased from the European Collection of Cell Cultures (ECACC, Salisbury, UK). The mouse lymphoma cell line L5178Y and resistant L5178MDR were donated by Dr. M. M. Gottesman (National Cancer Institute, Bethesda, MD, USA).

### 4.2. Preparation of Double-Loaded with Doxorubicin and Resveratrol Complex Composite Chitosan-Albumin Nanogel

The loaded composite nanogels were prepared at a molar ratio of 1:1 between the drugs via electrostatic gelation followed by a heating method [11] with a modification. Firstly, a complex between trans-resveratrol (1.25 mg) and hydroxypropyl-β-cyclodextrin at a ratio of 1:10 (wt./wt.) was obtained via solvent evaporation method according to previously described procedure [31]. Then, 3 mg of doxorubicin and 25 mg of bovine serum albumin were mixed with the complex dispersion. A 1 mL 0.5% solution of chitosan (*w*/*v*) in hydrochloric acid buffer (pH = 1.2) was dripped into the dispersion, and the mixture was stirred at 700 rpm for 90 min. Therefore, it was alkalized with a sufficient amount of 1M NaOH to a pH of approx. 4.6. The system was heated at 78 °C for 20 min and then stirred for 3 h at 700 rpm. After that, the dispersion was filtered (0.2 µm), the filter was rinsed with 50% ethanol, and the concentration of unloaded drugs in the filter fraction was determined spectrophotometrically at 480 nm for doxorubicin and 306 nm for resveratrol (Thermo Fisher Scientific, Waltham, MA, USA). Standard curves of doxorubicin were prepared in the range of 10–80 µg/mL in buffers with pH = 5 (r > 0.9997) and pH = 7.4 (r > 0.9991). A standard curve of resveratrol was prepared in 50% ethanol in the range of 2–10 µg/mL (r > 0.9996). The encapsulation efficiency (EE) and loading degree (LD) were determined according to the following equations:

EE = (Total amount of drug − Non-loaded drug)/Total amount of drug(1)

LD = (Total amount of drug − Non-loaded drug)/Volume of drug loaded micellar dispersion(2)

### 4.3. Physicochemical Characterization of the Loaded Nanogels

The mean diameter and polydispersity index of the systems were determined after 10 times dilution of the aqueous nanogel dispersion with purified water. The measurements were performed via photon correlation spectroscopy at a scattering angle of 90°. Phase analysis light scattering (PALS) method at a scattering angle of 15° was applied for evaluation of the zeta potential (Zetasizer NanoBrook 90Plus PALS, Brookhaven Instruments Corporation, Holtsville, NY, USA). The size and shape of the nanoparticles were confirmed via transmission electron microscopy (TEM, HR STEM JEOL JEM 2100, Tokyo, Japan). The diluted (1:10 in water) nanogel dispersion (approx. 5 µL) was pipetted onto a TEM grid and was allowed to dry at room temperature. The grid was directly observed in the TEM after the medium was evaporated.

Nicolet iS5 FTIR spectrometer (Thermo Fisher Scientific, Waltham, MA, USA), accumulating 64 scans at a spectral resolution of 2 cm^−1^, was used for collecting the IR spectra. The samples were prepared in KBr pellets.

Bruker D8 Advance diffractometer (Bruker Corporation, Billerica, MA, USA) with Cu Kα radiation and a LynxEye detector was used for recording the powder X-ray diffraction patterns of doxorubicin, trans-resveratrol, chitosan, albumin, and the loaded nanoparticles in the 5–80° 2θ range with a step of 0.02°. The samples were placed in a standard sample holder.

### 4.4. In Vitro Release Studies

In vitro release tests were conducted in two 10% ethanol buffer media with pH values of 7.4 (phosphate buffer) and 5.0 (citrate buffer) via dialysis method [63]. Nanogel dispersion (2 mL, containing 1.058 mg doxorubicin and 0.624 mg resveratrol) was introduced into a dialysis bag (10,000 MWCO, Spectrum Labs, San Francisco, CA, USA) and then placed in 40 mL of the media. The test was performed in a water bath at 37 °C under gentle shaking (IKA Labortechnik HS-B20, Staufen, Germany). Samples of 3 ml were taken at predetermined time intervals, and the same amount of fresh buffer was returned to maintain sink conditions. The concentrations of doxorubicin and trans-resveratrol were determined spectrophotometrically as described above.

The data from the release tests was fitted to zero-order (Equation (3)), first-order (Equation (4)) and Higuchi (Equation (5)) models in order to evaluate the release kinetics:

C_t_ = C_0_ + K_0_·t, (3)

where C_t_ is the amount of active substance released during the time t; C_0_ is the initial concentration of the drug released; and K_0_ is the zero-order rate constant.

ln(C_i_ − C_t_) = ln(C_i_) − K_1_·t, (4)

where C_t_ is the amount of active substance released during the time t; C_i_ is the initial concentration of the drug before release; and K_1_ is the first-order rate constant.

C_t_ = K_H_·t^1/2^, (5)

where C_t_ is the amount of drug released during the time t, and K_H_ is the release constant of Higuchi.

### 4.5. Cell Cytotoxicity Studies

The H9c2 and SH-SY5Y cells were maintained in Dulbecco’s Modified Eagle’s Medium and Roswell Park Memorial Institute 1640 Medium, respectively, which were supplemented with 10% fetal bovine serum and 4 mM L-glutamine. The L5178Y and L51L5178 MDR cells were maintained in McCoy’s 5A Medium, supplemented with 10% horse serum, 4 mM L-glutamine, and 60 ng/mL colchicine only for the MDR cell line. The cell lines were incubated under standard conditions (5% CO_2_, 37 °C, high humidity, Esco CelCulture^®^ CO_2_ Incubator, CCL-170B-8-IVF, Esco Micro Pte. Ltd., Singapore) and subcultured according to the protocols for adherent [64] and suspension [65] cell lines.

Cell cytotoxicity assays were conducted on rat cardioblast (H9c2) and neuroblastoma (SH-SY5Y) cells via neutral red assay [66] and on lymphoma L5178Y and L5178MDR cells via Alamar blue assay [67]. The cells were seeded in 96-well plates at a cell density of 0.5 × 10^4^ (H9c2), 2.5 × 10^4^ (SH-SY5Y) and 1 × 10^4^ (L5178Y and L5178MDR). Then, they were incubated overnight at standard conditions, namely 37 °C, 5% CO_2,_ and high humidity (Esco CelCulture^®^ CO2 Incubator, CCL-170B-8-IVF, Esco Micro Pte. Ltd., Singapore). Thereafter, the cells were treated with an aqueous solution of doxorubicin, double-loaded nanogel, and a non-encapsulated hydroalcoholic mixture of doxorubicin and resveratrol. The cardioblast and lymphoma cell lines were treated with 0.25 and 5 µM concentrations of doxorubicin and resveratrol, whereas the neuroblastoma cell line was with 0.125 and 0.25 µM. For H9c2 and SH-SY5Y cells, neutral red solution in the appropriate mediums (40 µg/mL) was added to each well (100 µL/well), and the plates were incubated for 3 h at 37 °C. Phosphate-buffered saline (PBS) was used for washing the cells, and then 100 µL of a destaining solution per well was added. The plates were rapidly shaken for 10 min, and the optical density was measured in a Synergy 2 microplate reader (BioTek Instruments, Inc., Highland Park, Winooski, VT, USA) at 540 nm. For L5178Y and L5178MDR, a resazurin solution in PBS at 44 µM final concentration was added to each well. The fluorescence at 540 nm was measured immediately after resazurin addition for baseline fluorescence evaluation and after 3 h of incubation for viability evaluation in the Synergy 2 microplate reader.

The cells were also photographed with an inverted light microscope (Optika XDS-2, Ponteranica, Italy) and digital camera (Optikam Pro 8LT—4083.18LT, Montreal, QC, Canada). In order to facilitate visual interpretation, the background effects in images were attenuated by applying a Bandpass Filter in Fiji software (version 1.54g) [68].

### 4.6. Statistical Analysis

All the experiments were conducted in triplicate, and the results are expressed as mean values ± SD. Statistical analysis was performed using GraphPad Prism 8 Software (Dotmatics, San Diego, CA, USA). The groups of cells treated with pure doxorubicin, the double-loaded nanogel, or the non-encapsulated mixture of doxorubicin and resveratrol were compared via multiple t-tests with Holm–Sidak correction.

## Figures and Tables

**Table 1:** Mean diameter, polydispersity index, and zeta potential of empty (NG) and double-loaded with doxorubicin and resveratrol nanogel (DOX/RES-NG). Mean values ± SD (*n* = 3).

Sample	Size, nm	PDI	ƺ-Potential, mV
NG	51 ± 6	0.241	+35.54
DOX/RES-NG	30 ± 4	0.188	+51.23

**Table 2:** Correlation coefficients (r^2^) for zero-order, first-order, and Higuchi models were calculated through kinetic analysis of in vitro release data for doxorubicin.

pH of the Medium	Zero Order	First Order	Higuchi Model
7.4	0.8451	0.9557	0.9737
5.0	0.8824	0.9973	0.9867

**Table 3:** Correlation coefficients (r^2^) for zero-order, first-order, and Higuchi models were calculated through kinetic analysis of in vitro release data for resveratrol.

pH of the Medium	Zero Order	First Order	Higuchi Model
7.4	0.8203	0.9707	0.9060
5.0	0.8824	0.9973	0.9867

## Data Availability

Data are contained within the article.
